# Small Intestinal Diverticulosis: A Rare Cause of Intestinal Perforation Revisited

**DOI:** 10.1155/2020/8891521

**Published:** 2020-10-21

**Authors:** Ahmed Faidh Ramzee, Mohamed H. Khalaf, Khalid Ahmed, Ejaz Latif, Noreddin Aribi, Nizar Bouchiba, Rajvir Singh, Ahmad Zarour

**Affiliations:** ^1^Department of Surgery, Hamad General Hospital, Doha 3050, Qatar; ^2^Department of Acute Care Surgery, Hamad General Hospital, Doha 3050, Qatar

## Abstract

Jejunoileal diverticulosis (JID) is a rare and nonspecific symptomatic disease. It is usually an acquired condition associated with false diverticula and integrated with colonic diverticulosis which can be diagnosed incidentally or later with complications. A sixty-nine-year-old male presented with sudden onset generalized abdominal pain. Computed tomography (CT) imaging was suggestive of ileal diverticulitis with localized perforation. The patient was treated conservatively with IV fluids and antibiotics and kept nil per orem for three days and discharged after symptoms subsided. The patient returned with a similar presentation but with a greater intensity. CT with oral contrast revealed evidence of distal ileal perforation. The terminal ileum was resected, and a double barrel ileostomy was created. Six months later, the stoma was reversed after resecting 50 cm of proximal terminal ileum which included all diverticula. The patient had a smooth postoperative recovery. Small bowel diverticulitis is generally managed conservatively unless the patient's clinical condition mandates urgent exploration. This report may add knowledge and lead to a change in clinical practice.

## 1. Introduction

Jejunoileal diverticular disease is rare with a reported incidence of 0.02–7.1% on imaging and 0.03–8.0% on autopsy. It is usually found in patients over the age of 40 and is more common in males (male/female ratio 2 : 1). Initially described by Somerling in 1794, jejunoileal diverticulosis is rarer than duodenal diverticulosis; however, it is associated with a fourfold higher risk of complications including diverticulitis, fistula formation, perforation, and hemorrhage [1, 2]. Most small bowel diverticulae are asymptomatic; however, almost 10% may go on to develop complications as described, and thus, a more aggressive surgical approach has been warranted in such cases [3].

However, given the advanced age of patients presenting with complicated jejunoileal diverticular disease, nonoperative treatment may be a feasible option depending on the clinical condition of the patient [3]. The current practice related to perforated small bowel diverticulitis is resection of the diseased bowel and primary anastomosis if the conditions allow [3]. We present a case of a 69-year-old male who presented with abdominal pain and was diagnosed with a computed tomography scan to have a localized ileal diverticular perforation and was initially managed conservatively and discharged. He subsequently returned with recurrent symptoms and was diagnosed with perforation requiring operative intervention in terms of a laparotomy, bowel resection, and stoma creation with reversal three months later. The patient had a smooth postoperative course, and the diagnosis was confirmed on histopathological examination.

## 2. Case Presentation

**Figure 1:**
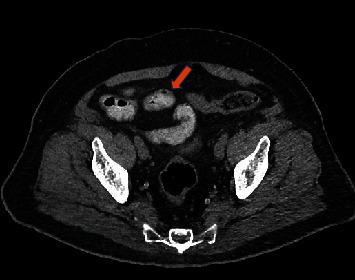
CT scan depicting focal wall thickening of the distal ileum and surrounding fat stranding and air loculi along the wall of the distal ileum (arrow).

A 69-year-old, diabetic, South Asian male presented to the emergency department of Hamad Medical Corporation (HMC) in October 2019 with sudden onset generalized abdominal pain more pronounced in the right lower quadrant and hypogastric region. He also had associated nausea and fever. On initial examination, he was afebrile and vitally stable and abdominal examination revealed tenderness in the right lower quadrant and suprapubic area with rebound tenderness. The remainder of his review of systems and physical exam was unremarkable. Computed tomographic (CT) examination of the abdomen showed multiple ileal diverticulae with focal wall thickening of the distal ileum and surrounding fat stranding and air loculi along the wall of the distal ileum suggestive of ileal diverticulitis with localized perforation (Figure 1). He was admitted and managed conservatively with IV fluids and antibiotics and kept nil per orem. His symptoms subsided, and he was discharged three days later.

**Figure 2:**
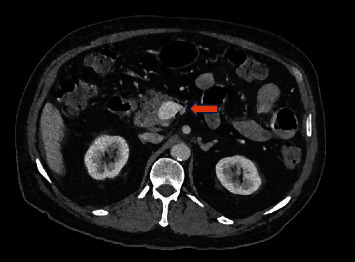
CT scan revealing perforation and collection at the distal ileum (arrow).

Two days after discharge, the patient returned to the emergency department with abdominal pain like the initial presentation but worse in intensity. A repeat CT with oral contrast revealed evidence of distal ileal perforation (Figure 2).

**Figure 3:**
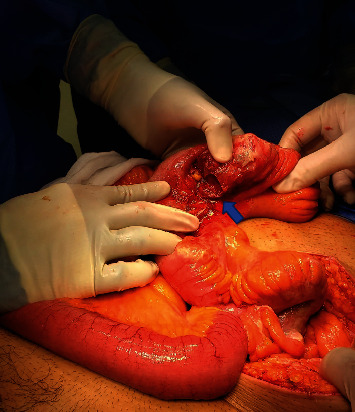
Intraoperative image depicting the site of ileal diverticular perforation on the mesenteric border.

**Figure 4:**
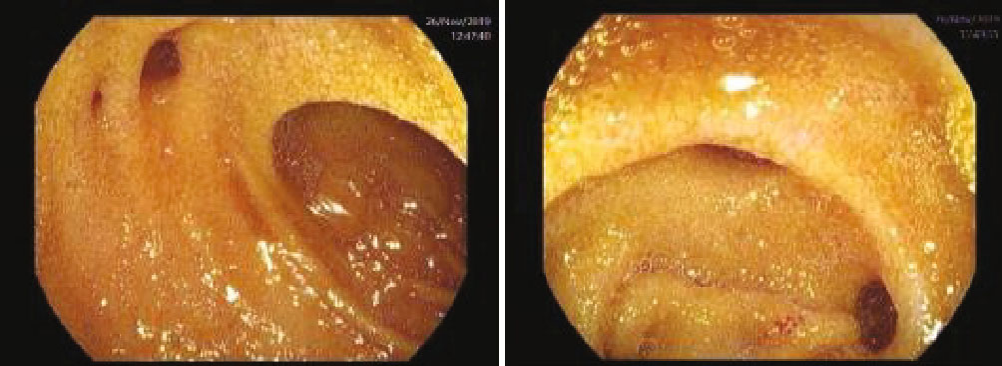
Postoperative enteroscopy depicting multiple diverticulae.

The patient was taken for a laparoscopic exploration, and extensive peritonitis was noted; therefore, the procedure was converted to a laparotomy. We noted a perforation in the ileum 50 cm from the ileocecal valve on the mesenteric aspect of the bowel with a purulent exudate covering the terminal ileum (Figure 3). The terminal ileum was resected, and a double-barrel ileostomy was created. His recovery was uneventful, and he was discharged on postoperative day 6. Histopathological examination of the resected bowel revealed 3 diverticulae with diverticulitis along with ulceration. Enteroscopic examination through the stoma showed multiple diverticulae up to 50 cm within the proximal limb (Figure 4). Three months postoperatively, his stoma was reversed after resecting 50 cm of proximal terminal ileum which included all diverticulae. Postoperative course was uneventful, and the patient made a full recovery. Final histopathology revealed diverticular disease in the resected portion of the ileum with no evidence of diverticulitis.

## 3. Review of Literature

**Figure 5:**
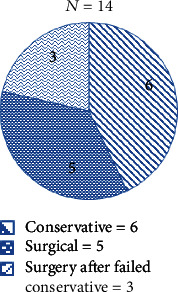
Pie chart depicting the mode of management for all cases.

The literature review yielded 14 cases of small intestinal diverticulitis presented in Table 1. All patients except two were above the age of 70 (age ranging from 29 to 87), the majority of whom were male (male to female ratio of 2.5 : 1). Four patients were operated on an emergency basis. Two patients underwent surgery due to the failure of conservative treatment (Case No. 8 and 10—Table 1). Three of the patients had complicated perforated diverticulitis and were successfully managed conservatively (Cases 3, 6, and 10—Table 1). One patient was managed conservatively and discharged but returned 2 months later with recurring symptoms and was operated upon, while another patient with recurrent symptoms was managed conservatively. Interestingly, one patient had recurrent presentation after having resection and anastomosis of jejunal diverticulitis a few years prior (Case No. 6—Table 1). The distribution of the type of treatment administered is displayed in Figure 1. None of the cases reported malignancy in the studied bowel specimens.  Figure 5 depicts the mode of management for all cases.

Lebert et al. also reported a multicenter retrospective study of 33 patients with jejunoileal diverticulitis over a 10-year duration. Most of whom were female (73 percent) with a median age of 78. Most patients had localized left side flank pain and elevations in inflammatory factors (leukocyte count and CRP). Thirty out of 33 patients had a positive CT scan, which detected an inflammatory diverticulum. They used Kaiser et al.'s modification of the Hinchey classification for acute diverticulitis and described peri-jejunoileal inflammation or phlegmon as stage IA, confined peri-jejunoileal abscess as stage IB, distant mesenteric abscess as stage II, and stage III as generalized purulent peritonitis. More than 70% of patients were stage IA. Eight patients who were found to have a severe presentation underwent emergent surgery. Conservative therapy was effective in 18 patients, all with moderate disease, and 5 patients underwent surgery after conservative treatment.

## 4. Discussion

Jejunoileal diverticulosis was first described over 200 years ago by Soemmering and Baille in their book titled *Anatomy of the Pathological Structure of Some of the Most Important Parts in the Human Body* in 1794 [2]. In contrast to large bowel diverticulae, those arising in the small bowel are quite uncommon, with the frequency of prevalence as well as the number of diverticulae descending from the duodenum (0.02% to 6%) to the jejunoileum (0.07% to 1%), with only 2.3-6.4% of these patients going on to develop diverticulitis [3, 4]. Most small bowel diverticulae produce no symptoms unless complicated by inflammation, perforation, bleeding, small bowel obstruction, or malabsorption [5]. Complications were noted to occur in approximately 10% of individuals, with jejunoileal diverticulae 3-4 times more likely to develop complications than duodenal diverticulae [6]. Mortality from perforated diverticulae is high, ranging from 21 to 40%; this has been attributed to the delay in diagnosis as well as the inherent risks associated with the elderly age of patients presenting with this disease [7]. Up to 60% of patients with small bowel diverticular disease may have concomitant colonic diverticulae [1].

Small bowel diverticulae are commonly seen in elderly males, in the sixth to seventh decade of life [8]. Acquired small bowel diverticulae are pseudo (false) diverticulae, consisting of a thin-walled outpouching formed by the mucosa and submucosa bulging through the muscular layer as opposed to Meckel's diverticulae (congenital) which contain all layers of the intestinal wall [1]. The pathogenesis has been explained to occur in areas of muscular weakness at the points of penetration of the vasa recta vessels along the mesenteric edge of the bowel [9].

There are no pathognomic signs or symptoms of ileal diverticulitis; hence, it needs a high index of suspicion. Patients may have diffuse acute abdominal pain or lower abdominal pain and tenderness with right lower quadrant features mimicking appendicitis. Fever and leukocytosis may also be commonly associated [10, 11]. Elderly individuals may pose a challenge in diagnosis due to a lack of clear physical signs; however, in rare circumstances, patients may present with subcutaneous emphysema due to intraperitoneal air causing rupture of the anterior abdominal wall which may help guide the clinician towards a diagnosis of intestinal perforation [12, 13].

The suggested method of radiological examination for diverticulitis is computed tomography. In a study where CT and ultrasound (US) of the abdomen was compared, CT was found to have a slightly greater ability to detect colonic diverticulitis than US [14]. Bowel gas may compromise US, therefore making detection of small bowel diverticulitis even more challenging. Ileal diverticulitis can mimic acute appendicitis; therefore, detection by CT abdomen has an additional benefit of the reduction in negative appendectomies, thereby preventing unnecessary surgery [1, 15]. CT with intravenous contrast is recommended [1, 16, 17]. The use of oral contrast in the treatment of patients with acute abdomen, however, is debated [1, 5].

Unlike the management of colonic diverticulitis, there is no grading system to stratify disease severity. The decision to proceed with conservative or surgical management is an area of controversy owing to the rarity of the condition and therefore a dearth of literature. It is generally accepted that any patient with perforated small intestinal diverticulae with generalized peritonitis and deterioration of the clinical status of the patient should undergo an operative segmental resection examination [7]. Diverticulae may be widespread throughout the intestine; therefore, the question arises as to how much length of the bowel needs to be resected and can we leave behind grossly normal diverticulae in order to avoid the risk of short bowel.

## 5. Conclusion

Non-Meckel's small intestinal diverticulitis is a rare entity and usually a disease of the elderly and thus carries a high potential for mortality. Delay in diagnosis may also increase the burden of morbidity and mortality. CT scans of the abdomen are the diagnostic modality of choice. However, in the presence of a negative CT and complicated patient symptomatology, diagnostic laparoscopy may be an acceptable option. The choice of conservative versus surgical management is a point of debate given that patients with complicated perforated diverticulae have been successfully managed conservatively. However, the risk of recurrence with a more severe presentation needs to be kept in mind as was the case in our patient. It is advisable to perform surgery for those patients with evidence of generalized peritonitis and deteriorating clinical parameters. Recurrent symptoms after conservative management may warrant surgical exploration depending on the clinical presentation of the patient. Resection of the affected segment of the bowel loop is the current standard keeping in mind the risk of short bowel and its associated concerns when determining the length of resection. Primary anastomosis may be performed if no doubts regarding bowel viability exist. Informed consent was obtained from the patient for publishing this case report.

## Figures and Tables

**Table 1:** Details of all 14 cases.

No.	Age	Sex	Symptoms	Signs	WBC-/L	Past medical history	Method of diagnosis and findings	Management	Histology	Follow-up	Ref.
1	82	M	LLQ and hypogastric pain	Diffuse tenderness	18.2		CT: jejunal diverticulitis with perforation	Surgical resection	Multiple jejunal diverticulae. No malignancy	Uneventful	[4]
2	48	F	RLQ pain	Localized tenderness	15.6		CT: thickening of the distal jejunal loop with extraluminal air bubbles	Surgery resection	Multiple jejunal inflamed diverticulae	NA	[4]
3	87	M	Abdominal pain & fever	Tenderness in LLQ	13.8		CT: small intestinal diverticulitis with perforation	Conservative		6 monthly follow-up for 5 years, no recurrence	[5]
4	78	F	Abdominal pain+diarrhea	NA	16.4	Hypertension, hyperlipidemia, atrial fibrillation, and diabetes. Osteosarcoma of the thigh with lung metastasis	CT: small intestine diverticulitis, with a large diverticulum (4.7 cm) near the jejunum	Conservative		Small bowel obstruction one year later, managed conservatively. Dead at 7 years-esophageal adenocarcinoma	[5]
5	76	M	Postprandial abdominal pain	NA	19.9	Right hemicolectomy for hepatic flexure adenocarcinoma one year prior	CT: multiple jejunal diverticulae with an inflammatory process	Conservative		8 months follow-up, no recurrence	[5]
6	87	M	Epigastric pain for one week, bloating, loose stools	RUQ guarding and tenderness	7.7	Segmental resection and anastomosis of perforated jejunal diverticulum 3 years prior. Colonic diverticulae	CT: localized perforation of the small bowel with multiple dilated loops of small bowel surrounding an area of marked soft tissue stranding with multiple small locules of gas	Conservative		No recurrence	[7]
7	35	M	RLQ pain	Guarding and tenderness in RLQ. Febrile	15.5	Ileal & colonic diverticulosis	CT: Sigmoid diverticulitis. Repeat CT on 2nd admission confirmed	Conservative. Readmitted 2 weeks later, managed conservatively.		Elective surgery performed later	[8]
8	73	F	Diffuse+lower abdominal pain	Diffuse tenderness, hypobowel sounds	5.8	LGI bleeding 3 months prior. Descending colon diverticulosis	Barium enema X-ray	Conservative for 10 days-laparotomy-right hemicolectomy due to suspicion of cancer	Multiple diverticulae in the terminal ileum, one perforated. No malignancy	Died post-op day 8-acute myocardial ischemia. Autopsy-multiple small bowel diverticulae, not inflamed	[10]
9	29	M	Right-sided lower abdominal pain	Diffuse guarding and rigidity, more on the RLQ. Febrile	23	Recently diagnosed renal disease	Diagnostic laparoscopy	Diagnostic laparoscopy converted to laparotomy due to adhesions. Resection and anastomosis	Single inflamed diverticulum. No malignancy		[18]
10	81	M	Right lower abdominal painful mass	Tender mass in the right flank	13.9	Significant weight loss	CT: cavitated thin-walled lesion in RIF	Conservative till day 6; laparotomy with resection and anastomosis	Multiple jejunal diverticula with mucosal ulceration and inflammatory lesions	Discharged on day 5 post-op	[19]
11	79	M	Diffuse abdominal pain	Generalized tenderness with signs of peritonitis	16	Hypertension, chronic obstructive pulmonary disease, diabetes, and cholecystectomy	CT: thickening of the distal jejunal loop and thickening and infiltration of the mesenteric fat and free air in the mesentery	Surgical resection and anastomosis	Multiple jejunal diverticuale. No malignancy		[20]
12	67	M	Abdominal pain	NA	12.2	Colonic diverticulosis and an episode of gastrointestinal bleeding one year before	CT: colonic diverticulosis. Multiple diverticula of the small intestine, with signs of inflammation	Initially conservative. Presented 2 months later with recurrence, managed with surgical resection underwent double enterectomy	No evidence of malignancy	NA	[21]
13	77	M	Localized abdominal pain	RLQ tenderness	11.4	Gunshot wound to the abdomen requiring an exploratory laparotomy	CT: focally thickened loop of small bowel in the anterior midabdomen with a small collection adjacent to the thickened small bowel measuring 2.8 cm × 1 cm	Conservative		Well after 1 year follow-up	[22]
14	82	F	Generalized abdominal pain for one day	Generalized abdominal tenderness with signs of peritonitis	18.2		CT: multiple small bowel diverticulae with surrounding pockets of free air adjacent to the jejunal diverticula	Laparotomy-2 pin hole perforations-primary closure	NA		[23]

WBC: white blood cell count; NA: not available; RUQ: right upper quadrant; RLQ: right lower quadrant; LLQ: left lower quadrant; CT: computed tomography; references: [4, 5, 7, 8, 10, 16–21].
